# A qualitative study of the IPS employment specialist role in the context of Nav employment in Norway

**DOI:** 10.1186/s12888-025-06991-1

**Published:** 2025-05-23

**Authors:** Cathrine Moe, Beate Brinchmann, Unni Kolstad, Kristine Steen, David McDaid, Eoin Killackey, Miles Rinaldi, Marit Borg, Arnstein Mykletun

**Affiliations:** 1 Faculty of Nursing and Health Sciences, Nord University, Bodø, Norway; 2 Centre for Work and Mental Health, Nordland Hospital Trust, Bodø, Norway; 3 University of South-Eastern Norway, Drammen, Norway; 4 Department of Community Medicine, UiT – The Arctic University of Norway, Tromsø, Norway; 5 Centre for Research and Education in Forensic Psychiatry, Haukeland University Hospital, Bergen, Norway; 6 Division for Health Sciences, Norwegian Institute of Public Health, Oslo, Norway; 7 Care Policy and Evaluation Centre, Department of Health Policy, London School of Economics and Political Science, London, UK; 8 Orygen, Melbourne, Australia; 9 Centre for Youth Mental Health, The University of Melbourne, Melbourne, Australia

**Keywords:** Employment specialist, Individual placement and support, Mental illness, Rurality, Supported employment, Vocational rehabilitation, Work

## Abstract

**Background:**

Employment is recognised as a fundamental human right. Still, many people experiencing severe mental illness are outside the workforce. Appropriate employment has several benefits for mental health and is central to recovery and citizenship. Individual Placement and Support (IPS) integrates treatment and employment support and is an evidence-based model for supporting people experiencing severe mental illness to gain and maintain employment. Employment specialists are front-line workers of IPS. In Norway, the implementation of IPS is in a later phase and employment specialists are employed outside health services. This study explores and describes employment specialists’ job situation within this new context.

**Methods:**

Qualitative data were collected through field notes and five focus group interviews. The study participants were 36 IPS employment specialists located at 13 different sites in Northern Norway. Transcripts and field notes were analysed by thematic analyses.

**Results:**

Our findings show that the IPS structures are settled in Norway, but some challenges remain. The most prominent consequence of the new context is the challenge of integration within health teams. Nonetheless, employment specialists find their work with clients meaningful and having great impact with opportunities for personal and professional development.

**Conclusion:**

IPS is anchored in Norwegian policy and several of the early intervention challenges are resolved. Our study provides increased understanding of the employment specialists job situation within the new IPS context in Norway. Employment specialists are “front-line-workers” in enacting the IPS principles, and their perspectives on the contextual change are crucial in the development of IPS.

## Background

Even though work is recognised as a fundamental human right [1], many people experiencing severe mental illness remain outside the workforce [2]. Appropriate employment has several benefits for mental health and is central to recovery and citizenship [3, 4]. Individual Placement and Support (IPS) has been described as a paradigm shift in supporting people experiencing mental illness to gain and retain employment [5]. IPS was developed in US in the late 1990 s. The most significant change in practice was the integration of employment specialists into clinical health teams instead of job support being delivered by actors outside mental health services [6].

The effectiveness of IPS is thoroughly documented in Cochrane reviews [7–9] and meta‐analyses [10–13]. There is also a strong economic case for implementing IPS [14]. Non-vocational outcomes of IPS such as increased confidence, positivity, hope, level of functioning and quality of life are reported [15–17]. Nevertheless, challenges of implementation and sustainability in mainstream practice is well-known [18–20].

The IPS approach is based on eight principles: 1. Focus on competitive employment, 2. Eligibility based on client choice, 3. Integration of employment support and mental health services, 4. Attention to client preferences, 5. Personalised benefit counselling, 6. Rapid job search, 7. Systematic job development, and 8. Time-unlimited individualised support [21]. These principles are enacted through an IPS team. The core actors in such a team are IPS employment specialists, health service clinicians and employment and benefit advisors. Since the development in late 1990 s, IPS has expanded across the world in countries with diverse sociocultural conditions, labour laws, welfare systems and economic conditions [22]. The delivery of IPS will therefore vary in different welfare systems. IPS employment specialists have key roles in implementing IPS and enacting the IPS approach in supporting clients to find a job in a competitive working environment [23]. The employment specialist role is to coordinate vocational plans with all actors in the IPS team and employers in the local labour markets [24–28]. Once an IPS participant secures employment, the employment specialist maintains job support for as long as needed. IPS supervisors are responsible for training, supervision and evaluation of the employment specialists’ practice [23]. The role of employment specialists does not require a professional education but courses for training in the IPS approach have been developed in several countries.

Earlier research has explored the key competencies desired in employment specialists [29, 30] and their perspectives on the facilitators and barriers to employment for individuals experiencing mental illness [31–33]. In addition to being in the front-line of a paradigm shift “Navigating between unpredictable icebergs” [24], the employment specialists have also been “pioneers” in the development of the new service in the early implementation phase [23] and they see themselves as culture brokers between mental health services and the business world [33]. The complex and many facetted roles might partly be the reason why there is a high turnover of employment specialists [23, 34, 35].

The present study took place in Norway, a country characterised by a generous welfare system. Health and vocational services originate from two distinct sectors, each governed by separate legislation and funded independently. Norwegian mental health services offer both community-based care and specialised hospital treatment. The Norwegian Labour and Welfare Administration (Nav) delivers social and vocational services, along with social welfare benefits. Nav offices serve as Norway’s Public Employment Services, defined at the EU level as “the authorities that connect jobseekers with employers” ([36], para 6).

Since 2022, there has been a change in the formal organisation of IPS in Norway. IPS was first introduced in Norway in 2012, and started as a shared responsibility between health services and Nav. IPS employment specialists were often employed at Nav in time-limited positions, but had their daily work located in health teams. IPS was integrated into mental health treatment (with local variations) [23]. Gradually, IPS has developed from being a vocational rehabilitation intervention, to becoming a mainstreamed welfare employment scheme at Nav [37]. This involved formal organisational decision from the Health Directorate for all IPS employment specialists to be employed at Nav [38]. The decision states that the IPS employment specialists are not considered health personnel and thereby do not have access to clinical health records and patients’ health information (only based on patient consent). This contextual change in IPS is described in a new status report on IPS in Norway [In Norwegian] [39].

IPS has systematically been studied in the Norwegian context since 2012 [40]. Still, the new contextual situation generates a need for further analysis of the implications for practice [37]. Existing studies looking at employment specialists’ practice in Norway were all conducted in earlier implementation phases before the 2022 reform [23, 34]. The aim of this study is therefore to explore and describe the IPS employment specialists’ job situation since the implementation of the new IPS organisational context.

## Methods

This qualitative study was carried out by a research team consisting of experienced IPS researchers and advisors. The members of the team are both men and women and have various professional background (psychologists, nurses, occupational therapist, economy and public health), various clinical experience (mostly from mental health) and research competence within a broad range of research methods and subject areas. The research team was responsible for all stages of the research process, including discussions of methods of data collection, development of interview-guide, recruitment of participants, data analysis and dissemination.

### Contextual background for the study

This study is a part of the IPS implementation and research project IPSNOR [23]. The context of this study is Northern Norway, characterised by rural areas and long distances between towns and smaller settlements. A detailed description of IPSNOR and the Northern Norway context is presented in Moe et al. [23]. The first IPS team was established in Northern Norway in 2012, and the newest team established in 2023. At the time of data collection there were 15 IPS sites in Northern Norway, and 58 IPS employment specialists and IPS supervisors were employed by Nav. Five employment specialists were still employed in health services. Their job title has changed to “employment and education specialists”. The job description of the employment specialists did not change due to their employment situation, they were still following the guidance from the IPS manual. Still, the organisational frames were changed.

### Study participants and data collection

#### Participants

Study participants were 36 IPS employment specialists and supervisors participating in a Northern Norway seminar for IPS employment specialists. Most participants (n33) were employed by Nav. Eight participants had the role of IPS employment specialist supervisor, some of them had dual roles as both employment specialist and supervisor. Three employment specialists were employed in a mental health team, either a municipal team or a hospital outpatient service. The employment specialists had various professional backgrounds. We did not collect information of their background in the demographic information collection, but during interviews participants disclosed information of their professional background. Some had health or social care qualifications, previously being nurses or social workers, while others had qualifications and work experience from other sectors. 23 women and 13 men participated, with variations in age and work experience. Work experience as an employment specialist varied from four months to almost five years. Most participants had worked for around two years, meaning most participants had experienced the change in IPS context. A few employments specialists were only familiar with the new context. 13 IPS sites were represented meaning the participants worked in various local contexts. Some worked in rural areas, while others worked in small or middle-sized cities.

#### Data collection and data development

Data for this study were collected at the seminar. Data consist of notes from groupwork, field notes from plenary discussion and transcripts from five focus group-interviews.

##### Notes from groupwork and fieldnotes

During the seminar, the employment specialists worked in groups. They discussed factors describing and influencing their work situation and took notes. These notes were collected by the research team and used as data. After the group work, there was a plenary discussion about the employment specialists’ job situation. CM, UK, KS and MB took fieldnotes during the plenary discussion. These notes are included in the data.

##### Focus group interviews

Five focus group interviews were conducted with 36 employment specialists, following the method of Krueger and Casey [41]. CM, BB, UK, KS and MB moderated one group each. An interview guide was prepared by all authors based on knowledge from previous studies and contextual changes. Questions asked during interviews were for instance: “Can you describe how IPS is organised at your site?”, “What works well and what doesn’t work so well?”, and “Do you like your work as an employment specialist?—Why/why not?” Participants were also encouraged to speak openly about their work experiences with IPS. After the focus group interviews, the five moderators of the groups met and shared their immediate reflections. These were written and constituted foundations for the analyses. The interviews were audio recorded. One interview lasted for 60 min, and the other four interviews lasted for 90 min. Altogether there were 15 pages of notes from group work and fieldnotes, and 109 pages of transcribed text from focus group interviews.

###  CM, BB, UK, KS and MBData analysis

Braun and Clarke`s method of thematic analysis [42–44] has guided the analysis. The focus group interviews were transcribed verbatim, fieldnotes and notes from group work collected. First, CM and MB familiarised themselves with the data and presented early reflections and analysis for the research team. Comments from the research team were written down and included in further analysis. In the next step, the transcripts and fieldnotes were carefully read and inductively coded relating to the scope of the study. We systematised the codes in themes and subthemes and adjusted after discussions within the research team. The analysis has involved an iterative back and forth process, moving between the entire data, the codes and the produced analysis [42–44]. To ensure reflexivity, we gathered the research team twice during the analysis process. We sought to critically reflect upon pre-understanding and personal beliefs by opening for several perspectives. We also revisited data several times during analysis process to ensure a coherent interpretation of the data. The interviews and analysis were conducted in Norwegian. Quotes were translated to English in the last analytic and writing phase.

## Results

During the thematic analyses, we developed four themes: 1. IPS structures are settled, but there are still organisational challenges, 2. Having a meaningful job, 3. Opportunities for personal and professional development, and 4. Balancing relational work, information flow and institutional boarders. We elaborate on the themes below.

### IPS structures are settled, but there are still organisational challenges

Employment specialists expressed that a lot of organisational structures related to IPS have been settled over the last couple of years in Norway, for example the clarification of IPS responsibility between Nav and health services. At the time of interviews, most employment specialists had permanent employment with Nav. There was an end to the time-limited, project-based positions. Most employment specialists were satisfied with the settled structures. However, they experienced some challenges, including finding it harder to involve clinicians in the job seeking process and being less integrated in mental health teams than previously: “It is very closed. They [the clinicians] do not talk about people that they don`t believe we have anything to do with. It is a bit of a shame that we don`t get a view of what they are doing [clinicians in meetings]. If we were there, we could have been involved in discussions on who could be involved in IPS”. They spoke of differences in the extent to which clinicians engaged in IPS: “There are some that we never get referrals from, and others use us a lot”.

However, even if the structures, roles and responsibility of IPS had been clarified, substantial differences in IPS delivery still existed between sites in Northern Norway. The differences discussed during interviews surprised the employment specialists and illustrated how individual factors still impacted IPS delivery. For example, some health team leaders were creative in finding ways to make the employment specialists feel integrated despite the new national regulations “At our site everything works well. We have work desks, cars and telephones available both at Nav and in health teams.” So, despite the restrictions concerning patient data, the team tried to make the employment specialists feel integrated. Other leaders followed the regulations strictly and kept the employment specialists out of areas in which clients were discussed: “Because nothing is being discussed anonymously, employment specialists are taken out of meetings. We have people in my small team who don`t attend any joint meetings with the health team they are part of.” Besides not being able to argue for employment support for clients, a result of not being integrated in health teams was the employment specialists continuous need to provide information on IPS, as an intervention and giving updates on IPS clients. Turnover of clinicians and less day-to-day contact meant that employment specialists experience a lack of knowledge of IPS in their collaborating health team clinicians: “There are often people saying: IPS? What was that again? What are you doing?”. Even if IPS had existed for years in Northern Norway, employment specialists reported that they had to invest a lot of effort into continually informing clinicians about IPS.

Employment specialists were satisfied by being employed by Nav, but Nav was felt to be a bureaucratic organisation with established rules that were not always compatible with the flexibility characterising IPS: “What made IPS special is gradually picked away”. An example of Nav rules from some sites were use of cars in their daily work: “We used to drive cars in this job earlier, but now it has stopped [due to Nav rules]. First, we had to use taxis, then taking a taxi stopped because it was too expensive”. On the question of what they did then, one employment specialist answered: “We vary. Often, we use our own cars and write travel expenses. We bring clients even if we probably shouldn`t. We must do it to make the ends meet”.

There were concerns about the employment specialists’ integration into the organisational structure at Nav. Nav have employment specialists in other employment schemes such as those working with “traditional” supported employment (without integration with health services). Employments specialists in different schemes have different models guiding their practice. Some IPS employment specialists expressed feeing a loneliness in Nav even if they were organised together with other employment specialists. They emphasised the value of being in a team consisting of other IPS employment specialists for their group belonging and job satisfaction.

### Having a meaningful job

Despite institutional challenges, the employment specialists highlighted experiences of having a meaningful job with great impacts. The impacts mentioned were particularly on the lives of clients and for society.

Employment specialists emphasised the meaningful partnerships (relational work) with clients. Being able to work closely with a person over time gave space for creativity and possibilities for tailoring interventions to individual needs. Employment specialists used a variety of strategies for building relationships with clients “We can go for walks outside or take a drive. We talk about everything to get to know each other”. During interviews, employment specialists expressed the value of seeing how IPS impacted clients’ life by observing mastering, personal growth, and increased self-confidence throughout the IPS process “It is meaningful to have this job. I get to follow people throughout their recovery process, it is not just a scheme having a start and end”. They also explained how they could observe having impacted clients’ lives: “The reward and what makes me enjoy it [the job] are that sometimes you reach a point after weeks, months, maybe years, where the client gets a light in his/her eyes and starts to believe in the process. The body language changes, and you realise that a process is taking place. I’ve chosen to cherish those moments, because that’s what drives us to keep going and find good solutions”.

Employment specialists also experienced their work to be of great value for society. They referred to the Norwegian welfare state needing to reduce the number of people being outside the workforce and on health-related welfare benefits. The focus of increasing the employment rate has been high on the Norwegian national policy agenda, and the employment specialists were thereby sure of their work being in line with national policies. By contributing to increasing participation in the labour force and reducing costs related to health benefits they could make a societal impact. “Earlier the focus was on ensuring the clients disability benefits [in Nav], now we focus on the healthy side of people”.

### Opportunities for personal and professional development

The position as an employment specialist gave a range of developmental possibilities, both personal and professional. Employment specialists spoke of a variation in work burden throughout the various sites. For some, their workdays were stressful with fully booked schedules. For others, their days could be quite calm with little activity. Still, regardless of their work burden employment specialists’ experienced possibilities for personal growth and development. Employment specialists talked about their daily challenges related to be courageous and fearless in meetings with collaborators such as clinicians or employers. They needed to be persistent in knocking on doors and making phone calls “We must dare to work outwards and contact people we haven’t met before. And we need to enjoy being on the move”. They also expressed how they developed personal stamina in motivating job seekers to believe in themselves and believe they could succeed in getting a job.

The professional development was about becoming comfortable in their role as employment specialists, to get clarity of what was expected from them and from their collaborators. Experienced employment specialists expressed security in their role and tasks. During interviews they described how they now managed to verbalise their expectations towards clients, employers and clinicians: “We need to be clear from the first meeting. We must say that no one *gets* a job, everyone must make an effort”. For the newer employment specialists, their role and tasks were still unclear. Their experiences of receiving sufficient training varied. For those starting in a well-established team, it was easier to enter the role of an employment specialist than for those starting at a new team: “We need training from the beginning, we cannot find out everything ourselves”. For training, most employment specialists completed a national course in IPS (e-learning from 2022). This course was referred to as useful, but overarching and general, and everyone had to transfer the overarching guidelines into local practices. Employment specialists asked for national instructions on how to do their job: “There should be a shared understanding and management decisions concerning how it should be, and everyone should perform the job equally. But this is not the case. There are local variations”.

### Balancing relational work, information flow and institutional boarders

One result of the new IPS context was clearer institutional boarders. Navigating different institutional regulations, systems and routines, as well as information from various actors and expectations from the IPS fidelity manual, was a huge balancing act for the employment specialists. They could experience dilemmas in carrying information that they believed collaborating partners should have access to, but due to confidentiality employment specialists could not share “Nav information” with health clinicians and vice versa. Moreover, information to employers were steered by the client. These dilemmas of carrying information were especially prominent if clients experienced substance misuse or had a criminal background. The burden of knowing without being able to tell employers was hard for some: “For me, it is a burden not to be able to tell the employer”. Employment specialists reflected upon their responsibilities due to openness: “Person 1: They [the employers] cannot demand more openness for IPS jobseekers than for the man on the street who went into the same interview and said nothing about own problems. Person 2: I agree, we all know someone who experience substance misuse and still manage their job. But then it is their responsibility. What if a person did something that harmed another person, and I knew that I could have stopped it [by being open]”. The issue of openness was especially challenging in small communities. Even if clients chose not to be open, their history of substance misuse or crime could exist ahead of them. Rumours and old narratives of clients could exist in small communities despite of clients’ actual life situations. Therefore, employments specialists navigated various strategies for information flow and openness, and still stayed focused on creating a trusting relationship with clients. Another result of the lack of daily contact with clinicians, was that some employment specialists felt unsure if they got essential health information of clients in order to provide them with the best support.

Employment specialists also discussed the need of navigating both Nav and health service structures concerning opportunities for flexibility described in the IPS fidelity manual. They had to negotiate with clinicians on keeping clients in their clinical records over time. The time limited clinical treatment period in health services did not harmonise with the principle on unlimited IPS support. How long the clinicians kept clients in treatment depended on the type of treatment and health team regulations. For some teams, clinicians were pressured to discharge clients early. Other teams were more open to keeping the clients in treatment for a longer period to ensure IPS participation. Negotiating with clinicians and health team leaders was challenging, and employment specialists shared various strategies. “At our site we`re generous when it comes to treatment time. The clinicians realise that if they are going to have IPS, their clients need to be in treatment. So even though clients don’t need to be in treatment any longer, clinicians manage in a way to organise treatment to last longer. For example, to have longer time between meetings or a kind of drop-in until we are satisfied”. Employment specialists also expressed dilemmas in navigating various demands: “We needed to be clear for the leaders- if they want IPS, they need to keep clients for a long time”, but then another employment specialist replied: “We lost IPS referrals due to that”.

## Discussion

This study has explored and described the employment specialists job situation in the new IPS context in Norway. The new context is characterised by both a later implementation phase of IPS, and the national decision of employing employment specialists at Nav including the statement of not defining the employment specialists’ work as health care. We will structure the discussion relating to the two contextual situations.

A later implementation phase is characterised by a continuing and long-term application of the new intervention to reduce unjustified variability in clinical practice [45]. In our previous study of the employment specialists practice in the early implementation phase (removed for peer review), the interviews with employment specialists were characterised by their overwhelming implementation work. The employment specialists spent most of their time working on the establishment of the new service, figuring out where they should have their daily work, how to communicate with collaborators, and how to balance the claims from Nav, health services, clients and IPS fidelity review manual (removed for peer review). For this current study, the employment specialists practice was characterised by being stable. There was less focus on implementation and service development, which gave room for in-depth practice reflection. The employment specialists clearly agreed they have a meaningful job with great impacts, and they experienced possibilities for personal and professional development. The relational work with clients was highlighted as particularly meaningful, corresponding with the factors Butenko et al. [34] found nourishing for the job of employment specialists. Furthermore, Borowska et al. [16] emphasised the relationship between employment specialists and clients as essential in IPS. In previous studies, the complex role of the employment specialist has been highlighted [23, 24, 46]. The turnover rates of IPS employment specialists are significantly higher than in other public sector occupations [46] and Butenko et al. [34] revealed that the majority of employment specialists who quit their jobs in the early implementation phase did this due to contextual work environment problems. For those IPS employment specialists who work within settled stable organisational structures, they have room for focusing on the work with clients, indicating that some of the work problems during implementation are resolved [34]. Previous studies describe how employment specialists see themselves as culture brokers between mental health services and the business world, and how they navigate unpredictable fields [24, 33]. In a later implementation phase, this seems to have changed. The employment specialists practice is more predictable, and the institutional cultures have come closer. Nevertheless, now the employment specialists fight against closed doors in health services.

The employment specialists relational work with clients experiencing severe or moderate mental illness also bring dilemmas. As employment specialists did not necessarily have formal health or social qualifications, they were dependent on support and advice from health clinicians. Lack of daily contact with clinicians made some employment specialists feel they were on their own in supporting their clients and unsure if they had enough essential health information on clients regarding the job seeking process. These findings illustrate the need of the employment specialists for a supporting health team, as emphasised by the IPS model. Employment specialists’ integration into clinical teams is core in the IPS Fidelity manual [47]. The argument behind this decision is the integration of job seeking into treatment. Distancing the employment specialists from the clinical teams, may cause less support for employment specialists and undermines the integrated support model. It might also cause less attention on employment in treatment. The decision to separate the employment specialists work from health care has been taken by the Norwegian Health directorate. Still, new national governmental documents promote employment as central in health policies [48, 49]. Thereby, at first sight the health policies in Norway seem unclear concerning the role of health services in supporting patients to find and maintain employment. It is clear that employment is beneficial to mental health recovery [3, 4, 50] and the Norwegian Health directorate has therefore introduced a new role, “Employment and education specialist”, into health services. The employment and education specialists’ work aim for ordinary employment, education or other meaningful activity for patients, and their work is defined as health care [51]. This new role visualises that employment is important for people’s health, but it seem like Norwegian stakeholders strive to find ways to integrate services delivered from Nav and health services.

How Norwegian policy gradually developed IPS from vocational rehabilitation to an employment scheme has been presented by Moe et al. [37]. The long-term consequences of the Norwegian way of delivering IPS are still unclear. This study contributes to new insight by presenting consequences for the employment specialists’ job situation. We still do not know how this organisation of IPS will impact the job seekers. Before the organisational change in Norway, IPS job seekers stated that that IPS benefitted their everyday lives [16]. A risk of removing the employment specialists’ integration in health teams, can be the ‘creaming’ of IPS clients involving a selection of people experiencing less severe mental illness, due to “those who seem most likely to succeed in terms of bureaucratic success criteria” [37, 52]. The evidence of IPS is strongly related to the IPS manual. The statement of employing employment specialists at Nav has clearly led to a deterioration in integration with the health services. This might violate the original IPS principle of being integrated in health services [6], and there is a risk of the IPS fidelity scores being reduced. IPS delivered to fidelity is an evidence-based service, and we do not know the long-term impact on effectiveness. Changing such a fundamental premise in the IPS model may give reason to question whether the effectiveness of IPS on employment for the original target group will be maintained in Norway. Another concern is that it can place additional burden on employment specialists if they are required to integrate with health teams without support through legislative changes or national guidelines. That will leave the responsibility to local solutions and the employment specialists.

### Study strength and limitations

The strength of our study is the rich data from various sources. The study includes perspectives from employment specialists with various backgrounds working in different IPS sites. We can therefore provide increased understanding of consequences of the new IPS context in Norway. Our study also has several limitations. As the employment specialists were gathered at a seminar and in focus group interviews, we are not able to connect experiences to contextual issues, work experience, gender or professional background. Also, we cannot identify how group dynamics have influenced the study results or how the presence of the research team has influenced group work and plenary discussions.

### Implications for practice and research

IPS implementation has been successful in Norway in terms of the increase in the number of IPS services, and their continuing and long-term application in practice. Findings from this study have implications for IPS practice and research. Even though there are institutional and structural barriers to the integration of employment specialists in health teams, clinicians and their leaders should seek to find solutions for involving employment specialists within their institutional frames and regulations. Additionally, at Nav, space for flexibility should be promoted to ensure the feasibility of the IPS principles. Further, there is a need of facilitating the employment specialist job situation in the navigation between institutions. Our study has also implications outside Norway. As several countries implement IPS, the context in which IPS is provided will vary. Decision makers must take into account that IPS are introduced differently from where they were initially developed and tested and be aware of possible consequences on a longer sight. This also applies to other evidence- based models than IPS. The implications for practice should be explored by research. Our findings suggest further research on the implications for employment specialists’ job situation and IPS in general following the move to the new context in Norway. Further research should also explore ways to support employment specialists to counteract their high turnover rate, and the implications for IPS effectiveness following the change in practice.

## Conclusion

IPS is anchored in Norwegian policy and several of the early intervention challenges are resolved. Our study provides increased understanding of the employment specialists job situation following the new IPS context in Norway. Employment specialists are “front-line-workers” in enacting the IPS principles, and their perspectives on the contextual change are crucial in the development of IPS. Our findings show that the IPS structures are settled, but some challenges remain. The most prominent consequence of the new context is the challenge of integration with health teams. Still, the employment specialists find their work with clients meaningful and experience having a job with great impact.

## Data Availability

The datasets generated and analysed during the current study are not publicly available due to participant confidentiality but is available from the corresponding author on reasonable request.

## Abbreviations

IPS: Individual placement and support
Nav: The Norwegian Labour and Welfare Administration

## References

[1] United Nations. Universal declaration of human rights. 1948. https://www.un.org/en/about-us/universal-declaration-of-human-rights.

[2] OECD. Fitter minds, fitter jobs: from awareness to change in integrated mental health, skills and work policies, mental health and work. Paris: OECD Publishing; 2021. 10.1787/a0815d0f-en.

[3] Gammelgaard I, Christensen TN, Eplov LF, Jensen SB, Stenager E, Petersen KS. ‘I have potential’: experiences of recovery in the individual placement and support intervention. Int J Soc Psychiatry. 2017. 10.1177/0020764017708801. 28545319 10.1177/0020764017708801

[4] Perkins R, Repper J. Editorial. Recovery and the right to contribute. Ment Health Soc Incl. 2016;20:4.

[5] Corrigan PW. Recovery from schizophrenia and the role of evidence-based psychosocial interventions. Expert Rev of Neurotherapeutics. 2006. 10.1586/14737175.6.7.993. 10.1586/14737175.6.7.993 16831114

[6] Becker D, Drake R. A working life for people with severe mental illness. Oxford: Oxford University Press; 2003.

[7] Crowther R, Marshall M, Bond GR, Huxley P. Vocational rehabilitation for people with severe mental illness. Cochrane Database Syst Rev. 2001. 10.1002/14651858.CD003080. 11406069 10.1002/14651858.CD003080 PMC4170889

[8] Kinoshita Y, Furukawa TA, Kinoshita K, Honyashiki M, Omori IM, Marshall M, et al. Supported employment for adults with severe mental illness. Cochrane Database Syst Rev. 2013. 10.1002/14651858.cd008297.pub2. 24030739 10.1002/14651858.CD008297.pub2 PMC7433300

[9] Suijkerbuijk YB, Schaafsma FG, van Mechelen JC, Ojajärvi A, Corbière M, Anema JR. Interventions for obtaining and maintaining employment in adults with severe mental illness, a network meta-analysis. Cochrane Database Syst Rev. 2017. 10.1002/14651858.CD011867.pub2. 28898402 10.1002/14651858.CD011867.pub2 PMC6483771

[10] Brinchmann B, Widding-Havneraas T, Modini M, Rinaldi M, Moe CF, McDaid D, et al. A meta-regression of the impact of policy on the efficacy of individual placement and support. Acta Psychiatr Scand. 2020. 10.1111/acps.13129. 31733146 10.1111/acps.13129

[11] de Winter L, Couwenbergh C, van Weeghel J, Sanches S, Michon H, Bond G. Who benefits from individual placement and support? A meta-analysis. Epidemiol Psychiatr Sci. 2022. 10.1017/S2045796022000300. 35815640 10.1017/S2045796022000300 PMC9281491

[12] Metcalfe JD, Drake RE, Bond GR. Economic, labor, and regulatory moderators of the effect of individual placement and support among people with severe mentall illness: a systematic review and meta-analysis. Schizophr Bull. 2018. 10.1093/schbul/sbx132. 29036727 10.1093/schbul/sbx132 PMC5768052

[13] Modini M, Tan L, Brinchmann B, Wang M, Killackey E, Glozier N, et al. Supported employment for people with severe mental illness: systematic review and meta-analysis of the international evidence. Br J Psychiatry. 2016. 10.1192/bjp.bp.115.165092. 27103678 10.1192/bjp.bp.115.165092

[14] Park AL, Rinaldi M, Brinchmann B, Killackey E, Aars NAP, Mykletun A, McDaid D. Economic analyses of supported employment programmes for people with mental health conditions: a systematic review. Eur Psychiatry. 2022. 10.1192/j.eurpsy.2022.2309. 35983840 10.1192/j.eurpsy.2022.2309 PMC9491084

[15] Boland L, Kennedy M, Lynch LJ, Bonham-Corcoran M, Quinn S. Exploring the non-vocational outcomes of the individual placement and support (IPS) employment model. Irish J Occup Ther. 2024. 10.1108/IJOT-09-2023-0021.

[16] Borowska MA, Ørjasæter KB, Borg M, Stenvall B, Silbermann A, Rinaldi M, et al. “Without IPS I think I would really fall apart”: individual placement and support as experienced by people with mental illness-phenomenological peer research study. Qual Health Res. 2024. 10.1177/10497323241275046. 39428951 10.1177/10497323241275046 PMC12117130

[17] Wallstroem IG, Pedersen P, Christensen TN, Hellström L, Bojesen AB, Stenager E, et al. A systematic review of individual placement and support, employment, and personal and clinical recovery. Psychiatr Serv. 2021. 10.1176/appi.ps.202000070. 33940948 10.1176/appi.ps.202000070

[18] Markström U, Svensson B, Bergmark M, Hansson L, Bejerholm U. What influences a sustainable implementation of evidence-based interventions in community mental health services? Development and pilot testing of a tool for mapping core components. J Ment Health. 2018. 10.1080/09638237.2017.1417544. 29252043 10.1080/09638237.2017.1417544

[19] Noel VA, Bond GR, Drake RE, Becker DR, McHugo GJ, Swanson SJ, et al. Barriers and facilitators to sustainment of an evidence-based supported employment program. Adm Policy Ment Health. 2017. 10.1007/s10488-016-0778-6. 27891567 10.1007/s10488-016-0778-6

[20] Prior S, Maciver D, Aas RW, Kirsh B, Lexen A, van Niekerk L, et al. An enhanced individual placement and support (IPS) intervention based on the Model of Human Occupation (MOHO); a prospective cohort study. BMC Psychiatry. 2020. 10.1186/s12888-020-02745-3. 32641009 10.1186/s12888-020-02745-3 PMC7346406

[21] Bond GR, Drake RE. Making the case for IPS supported employment. Adm Policy Ment Health. 2014. 10.1007/s10488-012-0444-6. 23161326 10.1007/s10488-012-0444-6

[22] Bond GR, Drake RE, Becker DR. An update on individual placement and support. World Psychiatry. 2020. 10.1002/wps.20784. 32931093 10.1002/wps.20784 PMC7491619

[23] Moe C, Brinchmann B, Rasmussen L, Brandseth OL, McDaid D, Killackey E, et al. Implementing individual placement and support (IPS): the experiences of employment specialists in the early implementation phase of IPS in Northern Norway. The IPSNOR study. BMC Psychiatry. 2021. 10.1186/s12888-021-03644-x. 34930203 10.1186/s12888-021-03644-x PMC8690340

[24] Kinn LG, Costa M, Voll I, Austrheim G, Aas RW, Davidson L. “Navigating between unpredictable Icebergs”: a meta-ethnographic study of employment specialists’ contributions in providing job support for people with mental illness. J Occup Rehabil. 2021. 10.1007/s10926-020-09943-6. 33200260 10.1007/s10926-020-09943-6

[25] Drake RE, Bond GR, Goldman HH, Hogan MF, Karakus M. Individual placement and support services boost employment for people with serious mental illnesses, but funding is lacking. Health Aff (Millwood). 2016. 10.1377/hlthaff.2016.0001. 27269028 10.1377/hlthaff.2016.0001

[26] Rinaldi M, Perkins R, Glynn E, Montibeller T, Clenaghan M, Rutherford J. Individual placement and support: from research to practice. Adv Psychiatr Treat. 2008. 10.1192/apt.bp.107.003509.

[27] Drake RE, Becker DR. Individual placement and support: an evidence-based approach to supported employment (evidence based practice). Oxford: Oxford University Press; 2012.

[28] Rinaldi M, Perkins R. Implementing evidence-based supported employment. BJPsych Bulletin. 2007. 10.1192/pb.bp.106.010199.

[29] Corbière M, Brouwers E, Lanctôt N, van Weeghel J. Employment specialist competencies for supported employment programs. J Occup Rehabil. 2014. 10.1007/s10926-013-9482-5. 24114382 10.1007/s10926-013-9482-5

[30] Whitley R, Kostick KM, Bush PW. Desirable characteristics and competencies of supported employment specialists: an empirically-grounded framework. Adm Policy Ment Health Ment Health Serv Res. 2010. 10.1007/s10488-010-0297-9. 10.1007/s10488-010-0297-9 20229141

[31] Blitz CL, Mechanic D. Facilitators and barriers to employment among individuals with psychiatric disabilities: a job coach perspective. Work. 2006;26(4):407–19. 16788260

[32] Henry AD, Lucca AM. Facilitators and barriers to employment: the perspectives of people with psychiatric disabilities and employment service providers. Work. 2004;22(3):169–82. 15156083

[33] Moen EÅ, Walseth LT, Larsen IB. Experiences of participating in individual placement and support: a meta-ethnographic review and synthesis of qualitative studies. Scand J Caring Sci. 2020. 10.1111/scs.12848. 32271470 10.1111/scs.12848

[34] Butenko D, Rinaldi M, Moe CF, Brinchmann B, Wittlund S, Killackey E. What I thought was the dream job was a little different than I had expected”: a qualitative study exploring the turnover of IPS employment specialists’. J Vocat Rehabil. 2024;61:79–91.

[35] Vukadinc M, Schaafsma FG, Michon HWC, Maaker-Berkhof Md, Anema JR. Experiences with individual placement and support and employment – a qualitative study among clients and employment specialists. BMC Psychiatry. 2021. 10.1186/s12888-021-03178-2. 10.1186/s12888-021-03178-2 PMC8025385 33827498

[36] European Commission, (N.y). Employment, social affairs & inclusion. European Commission, European Network of Public Employment Services - Employment, Social Affairs & Inclusion - European Commission (europa.eu). Accessed 13 Aug 2024 from https://employment-social-affairs.ec.europa.eu/policies-and-activities/coordination-employment-and-social-policies/european-network-public-employment-services_en.

[37] Moe C, Brinchmann B, Borg M, McDaid D, Rinaldi M, Killackey E, Mykletun A. Implementing individual placement and support in Norway. From vocational rehabilitation to an employment scheme. Sos Policy Adm. 2022. 10.1111/spol.12881.

[38] Directorate of Health. Samarbeid mellom helse- og omsorgstjenesten og Nav om individuell jobbstøtte (IPS) – rettslig grunnlag for tverrsektorielt samarbeid. [Collaboration between Health- and careservice and Nav about Indivudal placement and support (IPS)- legal basis for cross-sectoral collaboration]. Letter to municipalities and Regional health service Trusts. 2022. Available from https://www.helsedirektoratet.no/tema/arbeid-og-helse/Samarbeid%20mellom%20helse-%20og%20omsorgstjenesten%20og%20NAV%20om%20individuell%20jobbst%C3%B8tte%20-%20IPS%20-%20rettslig%20grunnlag%20for%20tverrsektorielt%20samarbeid%20-%20brev%20270422.pdf/_/attachment/inline/dd8c6724-f907-4dd8-a819-0ebb58047cc7:b5fe272796f42367ff43da1397c5d95ebdc00b81/Samarbeid%20mellom%20helse-%20og%20omsorgstjenesten%20og%20NAV%20om%20individuell%20jobbst%C3%B8tte%20-%20IPS%20-%20rettslig%20grunnlag%20for%20tverrsektorielt%20samarbeid%20-%20brev%20270422.pdf. Accessed 07.02.2024.

[39] Proba samfunsanalyse. Status for individuell jobbstøtte (IPS) i Norge. Oslo: Proba samfunnsanalyse; 2024. Available from: Status for individuell jobbstøtte (IPS) i Norge – Proba samfunnsanalyse.

[40] Sveinsdottir V, Bull HC, Evensen S, Reme SE, Knutzen T, Lystad JU. A short history of individual placement and support in Norway. Psychiatr Rehabil J. 2020. 10.1037/prj0000366. 30945917 10.1037/prj0000366

[41] Krueger RA, Casey MA. Focus groups: a practical guide for applied research. Thousand Oaks: Sage; 2009.

[42] Braun V, Clarke V. Using thematic analysis in psychology. Qual Res Psychol. 2006. 10.1191/1478088706qp063oa.

[43] Braun V, Clarke V. Reflecting on reflexive thematic analysis. Qual Res in Sport Exerc Health. 2019. 10.1080/2159676X.2019.1628806.

[44] Braun V, Clarke V. Toward good practice in thematic analysis: Avoiding common problems and be(com)ing a *knowing* researcher. Int J Transgend Health. 2020. 10.1080/26895269.2022.2129597. 10.1080/26895269.2022.2129597 PMC9879167 36713144

[45] Tansella M, Thornicroft G. Implementation science: understanding the translation of evidence into practice. Br J Psychiatr. 2009. 10.1192/bjp.bp.109.065565. 10.1192/bjp.bp.109.065565 19794192

[46] Butenko D, Rinaldi M, Brinchmann B, Killackey E, Johnsen E, Mykletun A. Turnover of IPS employment specialists: rates and predictors. J Vocat Rehabil. 2020. 10.3233/JVR-221195.

[47] Becker DR, Swanson SJ, Reese SL, Bond GR, McLehman BM. Supported employment fidelity review manual. In: A companion guide to the evidence-based IPS supported employment Fidelity scale. 4th ed. 2019. https://www.centreformentalhealth.org.uk/sites/default/files/final-fidelity-manual-fourth-edition-112619_0.pdf.

[48] Helse- og omsorgsdepartementet. (2022–2023). Folkehelsemeldinga. Nasjonal strategi for utjamning av sosiale helseforskjeller. (Meld.St.15). Oslo. Retrieved from: https://www.regjeringen.no/no/dokumenter/meld.-st.-15-20222023/id2969572/?ch=1.

[49] Helse - og omsorgsdepartementet. (2022–2023). Opptrappingsplan for psykisk helse (2023–2033). Oslo. Retrieved from https://www.regjeringen.no/contentassets/0fb8e2f8f1ff4d40a522e3775a8b22bc/no/pdfs/stm202220230023000dddpdfs.pdf.

[50] Drake RE, Whitley R. Recovery and severe mental illness: description and analysis. Can J Psychiatry. 2014. 10.1177/070674371405900502. 25007276 10.1177/070674371405900502 PMC4079142

[51] Directorate of Health. Forklaring av rollen som arbeids/utdanningsspesialist [Explanation of the role as employment and education specialist]. 2023. Available from https://www.helsedirektoratet.no/tema/arbeid-og-helse/individuell-jobbstotte-ips/Forklaring%20av%20rollen%20som%20arbeids-%20utdanningsspesialist.pdf. Accessed 06.11.2024.

[52] Lipsky M. Street-level bureaucracy: dilemmas of the individual in public services, vol. 2. New York: Russel Sage Foundation; 2010.

